# Savanna Tree Seedlings are Physiologically Tolerant to Nighttime Freeze Events

**DOI:** 10.3389/fpls.2016.00046

**Published:** 2016-02-02

**Authors:** Kimberly O’Keefe, Jesse B. Nippert, Anthony M. Swemmer

**Affiliations:** ^1^Division of Biology, Kansas State UniversityManhattan, KS, USA; ^2^SAEON Ndlovu NodePhalaborwa, South Africa

**Keywords:** freezing, drought, savanna, hydraulic conductivity, gas exchange

## Abstract

Freeze events can be important disturbances in savanna ecosystems, yet the interactive effect of freezing with other environmental drivers on plant functioning is unknown. Here, we investigated physiological responses of South African tree seedlings to interactions of water availability and freezing temperatures. We grew widely distributed South African tree species (*Colophospermum mopane, Combretum apiculatum*, *Acacia nigrescens*, and *Cassia abbreviata*) under well-watered and water-limited conditions and exposed individuals to nighttime freeze events. Of the four species studied here, *C. mopane* was the most tolerant of lower water availability. However, all species were similarly tolerant to nighttime freezing and recovered within one week following the last freezing event. We also show that water limitation somewhat increased freezing tolerance in one of the species (*C. mopane*). Therefore, water limitation, but not freezing temperatures, may restrict the distribution of these species, although the interactions of these stressors may have species-specific impacts on plant physiology. Ultimately, we show that unique physiologies can exist among dominant species within communities and that combined stresses may play a currently unidentified role in driving the function of certain species within southern Africa.

## Introduction

Occasional freeze events can cause substantial die-back of dominant woody plants in tropical and subtropical savannas (Brando and Durigan, 2004; Holdo, 2005, 2006, 2007; Whitecross et al., 2012). Freeze events are generally uncommon in the savanna biome and savanna woody plants are considered to be more sensitive to frost than temperate species. If woody species in savannas are frost intolerant, then freezing temperatures can have three potential implications for the broad-scale distribution and ecology of savanna species. Firstly, regular frost may be an important factor that prevents savanna woody species from invading neighboring grasslands (Wakeling et al., 2012), particularly if freeze events kill seedlings of these species. Secondly, frost may restrict the distribution of dominant woody species within the savanna biome by preventing the expansion of tropical and sub-tropical dominants into savannas of higher latitude or altitude, where freeze events occur (Whitecross et al., 2012). Thirdly, in cooler savannas, occasional freeze events may limit the abundance of many woody species by limiting recruitment or “topkilling” adults, thus preventing dominance by woody plants and contributing to the co-existence of trees and grasses that characterizes these systems.

Despite the presumed importance of freezing in savannas, few studies have investigated the impacts of freezing temperatures on the physiology, abundance, or distribution of savanna trees, particularly among dominant species that contribute greatly to ecosystem structure and function. Furthermore, we lack an understanding of how multiple species within the same environment respond to the combined effects of freezing and other environmental drivers such as water availability (but see Holdo, 2005, 2006, 2007). Understanding how co-dominant species respond to interactions of freezing and water availability will be essential for predicting savanna responses to future climate change, particularly as the frequency of freezing increases and precipitation patterns become increasingly variable (New et al., 2006).

The semi-arid savannas of southern Africa illustrate ecological interactions of freezing and drought that likely impact the distribution of dominant woody species. In northeastern South Africa, two dominant and functionally distinct lowveld savannas span broad gradients in temperature and precipitation (Palgrave, 2002). The *Acacia–Combretum* savanna occurs in the cooler, wetter south and is co-dominated by species of the genera *Acacia and Combretum*, such as *Combretum apiculatum* Sond. (red bushwillow) and *Acacia nigrescens* Oliv. (knobthorn). Conversely, the Mopane savanna occurs in the warmer, drier north and is dominated by the ecologically and economically important subtropical tree *Colophospermum mopane* (Kirk ex Benth.) Kirk ex J. Léonard (mopane). Many tree species that occur in the *Acacia–Combretum* savannas, such as *Cassia abbreviata* Oliv. (sjambok pod), also occur in the Mopane savanna to the north, albeit at lower densities. However, *C. mopane* does not occur in the southern *Acacia–Combretum* savanna or areas where winter minimum temperatures drop below 5°C (Henning and White, 1974), suggesting that this species is more sensitive to freezing than species commonly found in the cooler, wetter *Acacia–Combretum* savanna (Whitecross et al., 2012).

While greater sensitivity to freeze events may restrict the southern distribution of *C. mopane*, frost may also play a role in excluding other common savanna tree species from neighboring grassland ecosystems that generally have colder winters (Wakeling et al., 2012). The effect of freeze events would have the greatest impact if these events drive mortality of seedlings, reducing population establishment. However, the impacts on juveniles or adult trees may also be significant if freeze events cause topkill and result in smaller individuals that are more susceptible to future frost (Whitecross et al., 2012), as well as subsequent disturbance events such as fire (Holdo, 2005). Despite broad correlations between climate and savanna tree distributions, the putative importance of frost and water availability on savanna tree physiology remains undocumented. Physiological research is needed to demonstrate if and how freezing affects savanna tree seedlings, particularly when occurring in combination with other environmental stressors such as drought.

Physiological responses to freezing and drought are closely linked because both stressors impair plant water status (Medeiros and Pockman, 2011). Like drought, freezing can disrupt hydraulic function and cause cellular dehydration (Verslues et al., 2006). During freeze events, water freezing in the xylem forces dissolved gasses out of solution (Utsumi et al., 1999) and when the water thaws the bubbles can expand and cavitate the vessel, reducing xylem hydraulic conductivity (Davis et al., 1999; Mayr and Sperry, 2010). Apoplastic freezing can also reduce plant water status because ice has a lower water potential than liquid water, which can force water out of the cell into extracellular space and cause both cellular dehydration and membrane structure damage (Pearce, 2001). Thus, strategies that confer drought tolerance such as osmotic adjustment (Morgan, 1984), altered cell membrane properties (Serrano et al., 2005), and small xylem vessel diameters (Tyree et al., 1994; Feild and Brodribb, 2001; Jacobsen et al., 2007; Charrier et al., 2014) are also associated with physiological cold tolerance in plants.

Although freezing and drought are functionally similar in their effect on plant-water relations, plant responses to interactions of these two stressors are difficult to predict because water availability can either reduce or exacerbate plant sensitivity to freezing, depending on how the freezing damage occurs. Plants can be more susceptible to freezing-induced cavitation under more negative xylem pressures (Sperry, 1995; Davis et al., 1999), suggesting that drought may exacerbate hydraulic freezing intolerance. Species that are drought tolerant and can sustain low xylem pressures may therefore experience greater cavitation during freeze-thaw events than less drought tolerant species. Conversely, drought may reduce cellular damage caused by freezing because water on a leaf surface can act as a nucleating agent (Wisniewski et al., 1997; Pearce, 2001) and because osmotic adjustment responses to water limitation may reduce cellular dehydration during apoplastic freezing (Xin and Browse, 2000).

Here, we assessed the physiological responses of four common woody savanna seedlings to freezing stress under water-saturated and water-limited conditions to better understand how these combined environmental drivers impact the dominant vegetation within South African savannas. Specifically, we addressed the following questions: (1) Does tolerance to water limitation vary among dominant savanna tree species? (2) Do these species differ in their sensitivity to freezing temperatures? (3) Does water availability modify physiological responses to freezing? Based on the distribution of these species in northeastern South Africa, we hypothesized that *C. mopane* seedlings will be more tolerant to water-limitation but more sensitive to freezing than species common in the cooler, wetter *Acacia–Combretum* savanna (*C. apiculatum*, *C. abbreviata*, and *A. nigrescens*). We also hypothesized that, if *C. mopane* is indeed more physiologically drought tolerant than the other species (e.g., if *C. mopane* can sustain lower xylem pressures than other species), water-limitation will exacerbate *C. mopane* responses to freezing relative to other species and freezing damage will occur primarily from loss of hydraulic function rather than cellular membrane damage.

## Materials and Methods

### Species and Growth Conditions

Seeds were germinated from four tree species native to southern Africa: *C. mopane*, *C. apiculatum*, *C. abbreviata*, and *A. nigrescens*. These species are widely distributed within southern African savannas, although the ranges of *C. apiculatum*, *C. abbreviata*, and *A. nigrescens* extend to lower latitudes and higher altitudes than that of *C. mopane*, including areas where freeze events are more likely (Palgrave, 2002). Seeds were germinated using the cultivation protocols of Venter and Venter (2007) in a greenhouse where temperatures were maintained between 20°C and 25°C daily. Following germination, seedlings were transplanted to 5 L treepots (1 individual per pot) (Stuewe and Sons, Tangent, OR, USA) and grown in a general-purpose growing medium (Pro-Mix BX Mycorrhizae, Hummert International, Topeka, KS, USA). Seedlings were watered as needed for 4 months to facilitate establishment and were fertilized once per week for the duration of the experiment with a commercially available fertilizer (MiracleGrow, Hummert International, Topeka, KS, USA). Pots were positioned randomly on greenhouse benches and were rotated each week to minimize the effect of microclimate variation on plant growth.

Four months after germination, all seedlings were split between two water treatments: “water-saturated” (watered to saturation every day) and “water-limited” (watered to saturation once per week). Water treatments were randomly assigned to individuals of each species and were maintained for the remainder of the experiment. The relative water content (RWC) of the soil in each pot was monitored in units of water fraction by volume (wfv) with a Hydra Probe II Soil Sensor (Sevens Water Monitoring System, Portland, OR, USA) every 2 weeks, immediately before the application of water to both the water-saturated and water-limited treatments.

### Pressure-Volume Curves

Pressure-volume curves were measured to assess leaf hydraulic traits associated with drought tolerance on five randomly selected individuals per species from the water-saturated treatment using the squeeze method, a variation of the bench-drying method (Tyree and Hammel, 1972). Plants were watered to saturation and placed in a dark growth chamber overnight to fully hydrate (leaf water potential > -0.5 MPa). The youngest, fully developed leaf from each individual was cut with a razor blade, wrapped in parafilm, and weighed on a microbalance (±0.1 mg; Ohaus Pioneer, Ohaus Corporation, Parsippany, NJ, USA). Leaf water potential was measured using a Scholander pressure chamber (PMS Instrument Company, Albany, OR, USA) and the pressure in the chamber was then increased by ∼0.2 MPa above the initial endpoint until water from the endpoint was completely evacuated. The leaf was re-weighed and the process was repeated until water could no longer be forced from the cut leaf surface. Following the final water potential measurement, the leaf was recut under water and allowed to fully rehydrate. An image was then taken of the leaf and leaf area was determined using ImageJ V1.48 (Rasband, 1997–2014). Leaf weight and the corresponding water potential were plotted to construct pressure-volume curves, which were used to identify the bulk leaf capacitance (*C_bulk_*), modulus of elasticity (ε), leaf water potential at turgor loss (π_tlp_), and osmotic potential at full turgor (π_0_).

### Assessing Physiological and Growth Responses to Water Availability

Physiological responses to water treatments were monitored on each species periodically for 5 months. Leaf-level gas exchange (CO_2_ assimilation at ambient C_a_, A_max_; stomatal conductance of water vapor, g_s_; and transpiration rate, *E*) was measured approximately every 4–5 weeks using an Li-6400xt open gas exchange system (Li-Cor, Inc., Lincoln, NE, USA) on 7–10 randomly selected individuals from each species in each water treatment. Gas exchange measurements were made on the youngest, fully developed leaf (*C. mopane* and *C. apiculatum*) or leaflet (*A. nigrescens* and *C. abbreviata*) of each plant and cuvette conditions were maintained at [CO_2_] = 400 μmol CO_2_ mol^-1^, relative humidity = 40–60%, and photosynthetically active radiation = 1500 μmol m^-2^s^-1^ photon flux density. Plants were allowed to stabilize for approximately 2–5 min in the cuvette and a single measurement was recorded. All physiological measurements were made between 10:00 and 14:00 h. Gas exchange calculations were adjusted for leaf area during data processing, if necessary.

Additionally, species growth responses to water treatments were monitored throughout the course of the experiment. Stem height and diameter were measured every 2 weeks on all individuals from each species in both water treatments.

### Nighttime Freezing Treatment

Nine-month-old seedlings were exposed to a sequence of nighttime freezing events to assess the sensitivity of each species to freezing temperatures. Due to spatial limitations inside the cold temperature chamber, one individual per species in each water treatment was randomly selected to receive the freezing sequence treatment and this freezing sequence was replicated over time on seven different groups of plants. Prior to freezing, each individual pot was covered in a plastic bag with holes to prevent edge effects in the freezing chamber. Additionally, ice chips were added to the soil to act as nucleating agents. At 20:00 h, all individuals in the block were transferred to a dark ESPEC ESU-3CA Platinous series environmental test chamber (ESPEC North America, Hudsonville, MI, USA) where the temperature in the chamber was gradually reduced to -5°C overnight. A minimum temperature of -5°C was chosen to reflect the absolute minimum temperatures experienced in South African lowveld frost events (Gertenbach, 1983; Kruger et al., 2002). The temperature in the freezing chamber began at 5°C and was slowly reduced to -5°C at a rate of 2°C/h, where it was maintained for 2 h before increasing again to 5°C at a rate of 2°C/h. At 07:00 h, the plants were returned to the greenhouse. This process was repeated twice more on the same plants, resulting in three consecutive nighttime freezing events per replicate group.

All measurements were made on each individual prior to the first nighttime freeze event (pre-freeze), following the first and third nighttime freeze events, and 1 week following the last freeze event (recovery). Although freezing-induced leaf mortality occurred throughout the freezing sequence, measurements were always made on the youngest, fully developed leaf still remaining on the plants. Leaf mortality due to freezing was assessed by determining the percent vegetation senesced on each individual 1 week following the last freeze event.

### Physiological Responses

Leaf gas exchange, midday leaf water potential, leaf hydraulic conductivity, chlorophyll fluorescence, and electrolyte leakage (EL) were measured on each individual during the pre-freeze, first freeze, third freeze, and recovery measurement periods. Leaf gas exchange (photosynthesis, stomatal conductance and transpiration) was measured as previously described and midday leaf water potential (Ψ_leaf_) was measured using a pressure chamber. The youngest, fully developed leaf (*C. mopane, C. apiculatum, and A. nigrescens*) or leaflet (*C. abbreviata*) from each plant was cut with a razor blade and placed in a dark, humidified polyethylene bag for approximately 1 h. After the equilibration period, leaf water potential (Ψ_leaf_) was measured using the pressure chamber. Photosynthetic efficiency (F_v_/F_m_, the maximum quantum efficiency of PSII) was measured with an Li-6400xt gas exchange system equipped with a fluorometer sensor head. Dark-adapted chlorophyll fluorescence was measured on morphologically and developmentally similar leaves at 07:00 h, after the cold treatment had ended but before the dark-adapted plants were returned to the greenhouse.

Leaf hydraulic conductivity was measured using the rehydration kinetics method (Brodribb et al., 2007). The youngest, fully developed leaf (*C. mopane, C. apiculatum, and A. nigrescens*) or leaflet (*C. abbreviata*) per individual was cut with a razor blade, sealed in a humidified plastic bag and placed in a cool, dark container for 1 h. A second leaf per individual was cut under water and allowed to rehydrate for 15–60 s, depending on the hydration status of the plant (typically 15 s). The rehydrated leaf was then sealed in a dark, humidified polyethylene bag for 1 h. Following the equilibrium period, water potential was measured on each leaf using a pressure chamber. Hydraulic conductivity was then calculated using equation 1:

$$K_{l\,e\,a\,f}=\frac{C_{b\,u\,l\,k\,\;\;}\,I\,n\,\;\;\frac{\Psi_{i\,n\,i\,t\,i\,a\,l}}{\Psi_{f\,i\,n\,a\,l}}}{t}\,\;\;\;\;\;\;\;\;\;\;\;\;\;\;\;\;\;\;\;\;\;\;\;\left(1\right)$$

where Ψ_initial_ is the water potential of the non-rehydrated leaf (MPa), Ψ_final_ is the water potential of the rehydrated leaf (MPa), *t* is the rehydration time (s), and *C_bulk_* is the bulk leaf capacitance measured from the initial slope of the pressure-volume curves for each species and normalized by leaf area (mmol m^-2^MPa^-1^).

Cell membrane damage resulting from the nighttime freezing treatment was assessed by the electrolyte leakage method (Wilner, 1960). Whole morphologically and developmentally similar leaves (*C. mopane* and *C. apiculatum*) or leaflets (*A. nigrescens* and *C. abbreviata*) were cut at the petiole with a razor blade, rinsed with distilled water, and placed in 50 ml test tubes filled with 25 ml distilled water. The tubes were shaken for 24 h at 20°C and 100 rpm, and then the conductivity of the solution was measured with an Oakton CON 510 electrical conductivity meter (Oakton Instruments, Vernon Hills, IL, USA). The tubes were then autoclaved for 30 min at 120°C and the final conductivity of the solution was measured. Electrolyte leakage was calculated as the ratio of conductivity before to after autoclaving (assuming that after autoclaving there is 100% electrolyte leakage).

### Statistics

Gas exchange physiology was compared among all species and water treatments prior to freezing using a three-way analysis of variance (ANOVA) in a completely randomized design with species, water treatment, and sampling date as fixed effects. Plant growth responses to water treatments and the soil RWC prior to freezing were compared using a linear mixed-effects model with species, water, and sampling date as main effects and plant as a random effect. Leaf parameters derived from pressure-volume curves were compared among species with a fixed effects one-way ANOVA in a completely randomized design. Physiological responses to freezing were assessed using a linear mixed-effects model with species, water treatment, and day (Pre-freeze, First freeze, Third Freeze, Recovery) as fixed effects, and plant and freezing sequence replicate as random effects. Leaf mortality was also assessed using a linear mixed-effects model with species and water treatment as fixed effects, and freezing sequence replicate as a random effect. Homogeneity of variances was assessed with Levene’s Test, all data were checked for normality, and multiple comparisons were calculated using Tukey’s Honestly Significant Difference test. All mixed effects analyses were conducted using the ‘lme4’ package V1.1-7 (Bates et al., 2014) and fixed effects analyses were conducted with the *‘lm’* function in the statistical program R V3.1.0 (R Core Team, 2012).

## Results

### Does Physiological Tolerance to Water Limitation Vary among Dominant Savanna Tree Species?

The RWC of the soil differed significantly among water treatments, species, and sampling date (*P* < 0.0001; Supplementary Tables S1 and S2). Soil in pots with the water-limited treatment had lower RWC values than soil in the water-saturated treatment, but the differences in water content reduction varied according to species (30–79%) (Supplementary Table S1). Soil RWC was typically higher in *C. mopane* and *C. apiculatum* than *A. nigrescens* and *C. abbreviata*. Additionally, RWC varied over time, but there was no increasing or decreasing trend throughout the experiment for any species and water treatment combination.

We assessed physiological drought tolerance using pressure-volume curves. Leaf parameters derived from pressure-volume curves differed significantly among some species (**Table 1**). The leaf water potential at turgor loss (*P* = 0.0002) and the osmotic potential at full turgor (*P* < 0.0001) were both significantly lower in *C. mopane* and *A. nigrescens* than *C. abbreviata* and *C. apiculatum*. *Colophospermum mopane* had the highest modulus of elasticity and this differed significantly from *C. abbreviata* and *C. apiculatum* (*P* = 0.0033). *Acacia nigrescens* had intermediate elasticity and did not differ from any other species. Bulk leaf capacitance did not differ between any species (*P* = 0.9476).

**Table 1:** Leaf parameters derived from pressure-volume curves and statistics assessing differences in parameters among species.

	*C_bulk_* (mmol m^-2^ MPa^-1^)	ε (MPa)	π_tlp_ (MPa)	π_0_ (MPa)
**Species**				
*C. mopane*	0.31 ± 0.05^A^	26.60 ± 2.04^A^	-2.22 ± 0.16^A^	-1.95 ± 0.14^A^
*A. nigrescens*	0.29 ± 0.03^A^	21.34 ± 2.11^AB^	-2.02 ± 0.05^A^	-1.76 ± 0.05^A^
*C. abbreviata*	0.27 ± 0.05^A^	14.22 ± 1.64^B^	-1.52 ± 0.08^B^	-1.12 ± 0.10^B^
*C. apiculatum*	0.31 ± 0.06^A^	15.73 ± 2.66^B^	-1.52 ± 0.08^B^	-1.10 ± 0.05^B^
**Statistics**				
*F*	0.12	6.95	12.59	21.61
*P*	0.9476	0.0033^∗^	0.0002^∗^	<0.0001^∗^

*Shown are mean ± 1 SEM bulk leaf capacitance (*C_*bulk*_*), modulus of elasticity (ε), leaf water potential at turgor loss (π_*tlp*_), and the osmotic potential at full turgor (π_*0*_). For each species, *n* = 5 water-saturated individuals. Superscripts indicate significant differences among species. *F* and *P*-values are calculated from a one-way ANOVA and significance is indicated at the α = 0.05 level with an asterisk (^∗^).*

**FIGURE 1:**
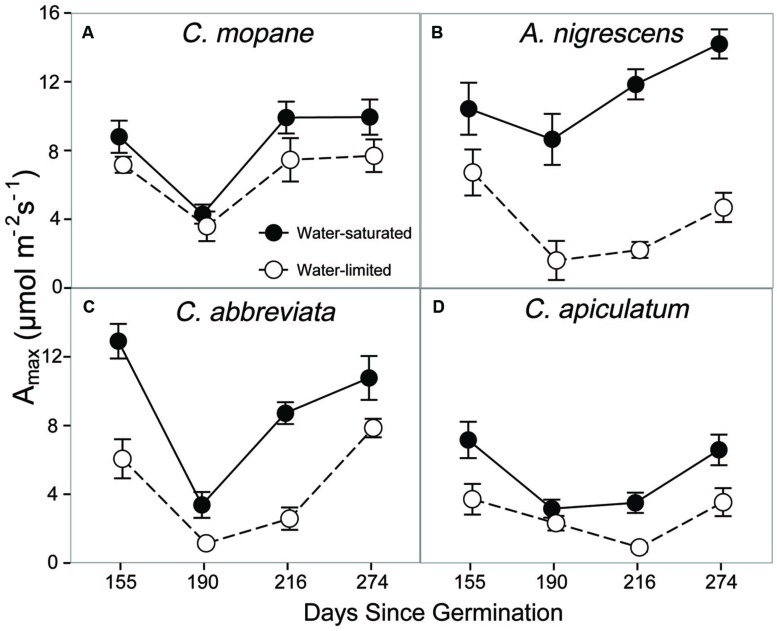
**CO_2_ assimilation at ambient C_a_, A_max_, measured prior to freezing in water-saturated and water-limited seedlings**. Shown are mean ± 1 SEM for **(A)** *C. mopane*, **(B)** *A. nigrescens*, **(C)** *C. abbreviata*, and **(D)** *C. apiculatum*. For each sampling date, *n* = 3–10 per species^∗^water treatment combination.

Additionally, we assessed physiological responses to water limitation by measuring leaf gas exchange on water-saturated and water-limited plants. Prior to freezing, leaf gas exchange varied among species, water treatments, and sampling date. We observed a significant species and water treatment interaction for photosynthesis (*P* < 0.0001; Supplementary Table S3). Photosynthetic rates were higher in water-saturated plants than plants that were water-limited, but this response was more pronounced in *A. nigrescens* and *C. abbreviata* than *C. mopane* and *C. apiculatum* (**Figure 1**). For instance, *A. nigrescens* photosynthetic rates were 66.8% higher in water-saturated plants compared to water-limited plants, but only 21.6% higher in water-saturated *C. mopane*. Generally, photosynthetic rates were lower in *C. apiculatum* than all other species, and water-saturated *A. nigrescens* had the highest rates. Similarly, we observed significant species and water treatment interactions in stomatal conductance (*P* < 0.0001) and transpiration (*P* < 0.0001) (Supplementary Table S3). *Acacia nigrescens* and *C. abbreviata* had greater stomatal conductance and transpiration rates when water-saturated, which also corresponded with larger difference in soil water content measured for these species compared to *C. mopane* and *C. apiculatum* (Supplementary Tables S1 and S4). Stomatal conductance (*P* = 0.0174) and transpiration rates (*P* < 0.0001) also varied over time; after an initial drop from the first to second sampling dates, rates continued to increase through the last sampling day (Supplementary Table S4).

Finally, we compared growth responses to water variability by measuring stem height and diameter on plants in both water treatments. Species, water treatments, and sample day were significantly interrelated in their effect on stem height (*P* < 0.0001) and stem diameter (*P* < 0.0001, Supplementary Table S2). Height and stem diameter were greatest in water-saturated plants, and *A. nigrescens* and *C. abbreviata* grew taller and had thicker stems than the other two species (Supplementary Table S5). *Colophospermum mopane* had the smallest reduction in height with water stress (9% compared to 12–36% for other species) and was the only species that did not suffer any reduction in stem diameter.

### Do these Species Differ in their Sensitivity to Freezing Temperatures and does Water Availability Modify Physiological Responses to Freezing?

We assessed leaf damage due to freezing by measuring leaf mortality 1 week following the last freeze event and found a significant interaction of species and water treatment (*P* = 0.0308, Supplementary Table S6). Leaf mortality due to freezing was lowest among water-saturated and water-limited *A. nigrescens* (10–15% mortality), and highest among water-saturated and water-limited *C. abbreviata* (52–65% mortality). Patterns of freezing mortality in *C. mopane* and *C. apiculatum* depended on water treatment. In *C. mopane*, water-saturated plants showed greater leaf mortality due to freezing than water-limited plants (57% compared to 20%, respectively), while the opposite response was observed in *C. apiculatum* (36% mortality in water-saturated plants versus 61% mortality in water-limited plants).

**FIGURE 2:**
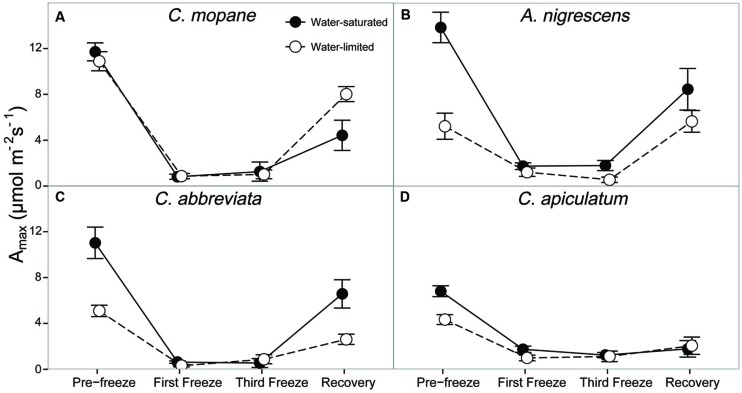
**CO_2_ assimilation at ambient C_a_, A_max_, measured across the freezing sequence in water-saturated and water-limited seedlings**. Shown are mean ± 1 SEM for **(A)** *C. mopane*, **(B)** *A. nigrescens*, **(C)** *C. abbreviata*, and **(D)** *C. apiculatum*. For each sampling day, *n* = 4–7 per species^∗^water treatment combination.

We also evaluated photosynthetic responses to freezing by measuring leaf gas exchange (photosynthesis, stomatal conductance, and transpiration), as well as dark-adapted chlorophyll fluorescence. Species, water treatment, and freezing event had a significant interactive effect on photosynthesis (*P* = 0.0006), stomatal conductance (*P* = 0.0035), and transpiration (*P* = 0.0063) throughout the freezing sequence (Supplementary Table S7). The first nighttime freezing event reduced gas exchange physiology similarly for all species and water treatments and these rates remained low until after the last freezing event (**Figure 2**; Supplementary Table S7). However, interactions among species and water treatments varied during the recovery period after the freezing sequence was complete. Photosynthetic rates were between 81 and 95% lower than initial rates for all species and water treatments by the last freezing event, but ranged from 40% (*A. nigrescens*) to 43% (*C. abbreviata*), 62% (*C. mopane*), and 74% (*C. apiculatum*) lower than initial rates 1 week later (**Figure 2**). Recovery photosynthetic rates also differed among water treatments, and the direction of this response varied among species. Water-saturated *A. nigrescens* and *C. abbreviata* had higher photosynthetic rates than water-limited plants, but water-saturated *C. mopane* had lower photosynthetic rates than water-limited plants and *C. apiculatum* recovery responses were similar among water treatments (**Figure 2**). Stomatal conductance and transpiration rates responded similarly to photosynthetic rates, both immediately after freezing and during recovery (Supplementary Table S7).

**FIGURE 3:**
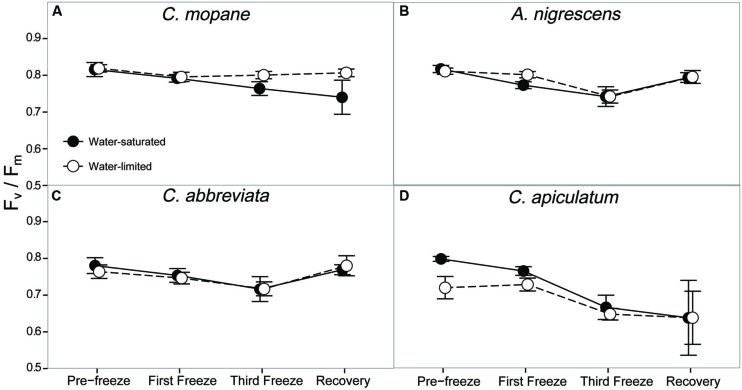
**Photosynthetic efficiency, F_v_/F_m_, measured across the freezing sequence in water-saturated and water-limited seedlings**. Shown are mean ± 1 SEM for **(A)** *C. mopane*, **(B)** *A. nigrescens*, **(C)** *C. abbreviata*, and **(D)** *C. apiculatum*. For each sampling day, *n* = 4–7 per species^∗^water treatment combination.

Dark-adapted chlorophyll fluorescence varied significantly between species and these differences varied across the freezing sequence (*P* = 0.0229; Supplementary Table S7). Averaged across all water treatments and freezing events, *A. nigrescens* and *C. mopane* had higher F_v_/F_m_ values than *C. abbreviata* and *C. apiculatum* (**Figure 3**). Nighttime freezing reduced F_v_/F_m_ by 10–20% of initial values in all species and water treatment combinations and this reduction was greatest in *C. apiculatum* during the recovery measurement (**Figure 3**).

**FIGURE 4:**
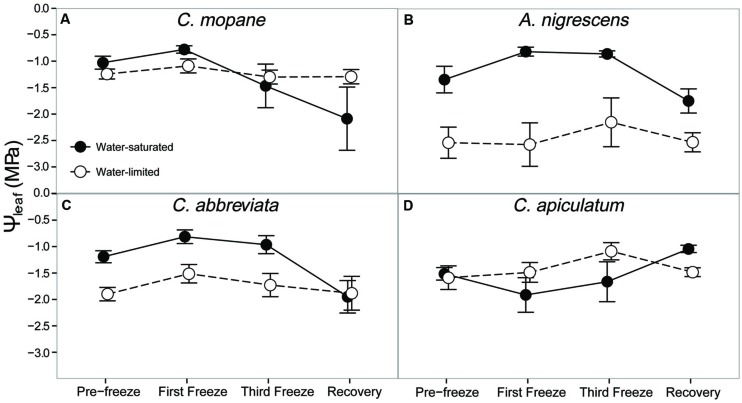
**Midday leaf water potential, Ψ_leaf_, measured across the freezing sequence in water-saturated and water-limited seedlings**. Shown are mean ± 1 SEM for **(A)** *C. mopane*, **(B)** *A. nigrescens*, **(C)** *C. abbreviata*, and **(D)** *C. apiculatum*. For each sampling day, *n* = 4–7 per species^∗^water treatment combination.

Additionally, we assessed hydraulic responses to freezing by measuring midday leaf water potential and leaf hydraulic conductivity. We observed significant species and water treatment (*P* = 0.0005) and species and freezing event (*P* = 0.0085) interactions on midday leaf water potential (Supplementary Table S7). In general, leaf water potential was lower in water-limited plants compared to water-saturated plants, although this difference was greatest in *A. nigrescens* compared to other species (**Figure 4**). Nighttime freezing reduced leaf water potential, but this also varied by species (**Figure 4**). After the first freeze event, leaf water potential increased slightly in each species, with the exception of water-saturated *C. apiculatum* and water-limited *A. nigrescens*. However, water potential values began to decline after the final freeze and remained lower than initial values during recovery. Of these species, *C. mopane* had the largest decline in leaf water potential by the recovery period (103% lower than initial values) and *A. nigrescens* had the smallest decline in leaf water potential (30% lower than initial values). Conversely, water potential declined slightly after the first freeze in *C. apiculatum* but then recovered 1 week after freezing. Water-limited plants were generally less responsive to freezing in all species, although this was not statistically significant (*P* = 0.0912).

**FIGURE 5:**
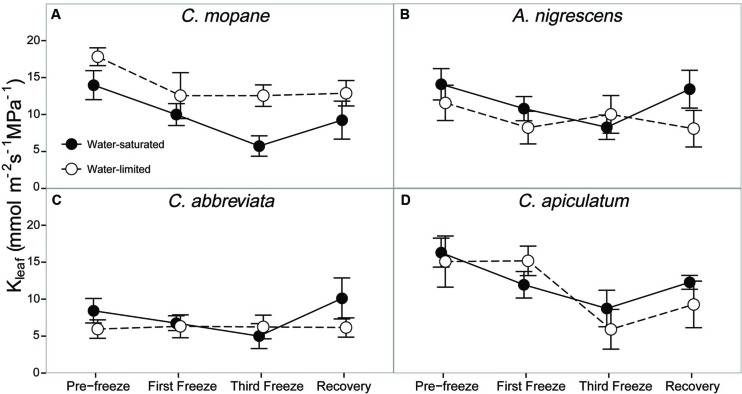
**Leaf hydraulic conductivity, K_leaf_, measured across the freezing sequence in water-saturated and water-limited seedlings**. Shown are mean ± 1 SEM for **(A)** *C. mopane*, **(B)** *A. nigrescens*, **(C)** *C. abbreviata*, and **(D)** *C. apiculatum*. For each sampling day, *n* = 4–7 per species^∗^water treatment combination.

Leaf hydraulic conductivity differed significantly among species (*P* < 0.0001) and freezing event (*P* < 0.0001; Supplementary Table S7). *Cassia abbreviata* had lower values than all other species and freezing decreased hydraulic conductivity similarly for all species and water treatment combinations (**Figure 5**). However, there was a significant interactive effect of species and water treatment on hydraulic conductivity (*P* = 0.0038). Hydraulic conductivity was similar among water treatments in all species except *C. mopane*, which had greater values in water-limited individuals.

**FIGURE 6:**
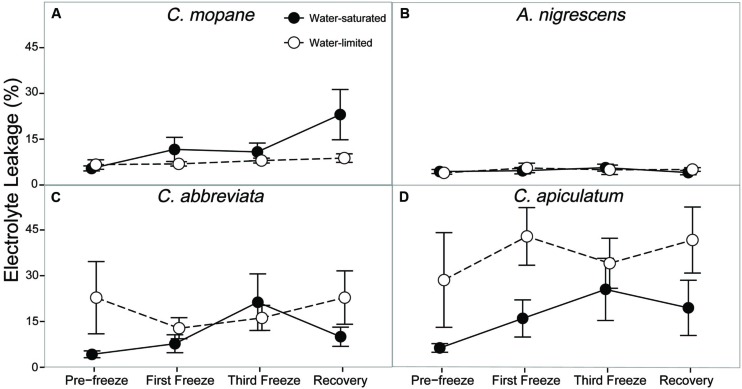
**Percent electrolyte leakage measured across the freezing sequence in water-saturated and water-limited seedlings**. Shown are mean ± 1 SEM for **(A)** *C. mopane*, **(B)** *A. nigrescens*, **(C)** *C. abbreviata*, and **(D)** *C. apiculatum*. For each sampling day, *n* = 4–7 per species^∗^water treatment combination.

Finally, we evaluated cell membrane damage caused by freezing by measuring percent electrolyte leakage. Electrolyte leakage differed among water treatments and this response varied among species (*P* = 0.0118; Supplementary Table S7). *Acacia nigrescens* had the lowest electrolyte leakage (generally less than 5%) and this was not affected by water stress or freezing (**Figure 6**). Conversely, *C. apiculatum* had the highest levels of electrolyte leakage and this was greater in the water-limited plants (37%) than the water-saturated plants (11%). Water-limited *C. abbreviata* also had greater electrolyte leakage than water-saturated plants, although this observation varied across sampling days. Unlike the other species, *C. mopane* had greater electrolyte leakage in water-saturated plants, and this difference between water treatments was most pronounced during recovery. Nighttime freezing also affected electrolyte leakage (*P* = 0.0347). Percent leakage increased from 10 to 25% in water-saturated *C. mopane* between the last freeze event and the recovery day. Water-saturated *C. abbreviata* also showed a large increase in electrolyte leakage, but between the first and third freeze events (10–22%); however, leakage was still similar or greater in water-limited plants across the freezing sequence for this species (**Figure 6**).

## Discussion

Nighttime freeze events can be important disturbances in savanna ecosystems, yet the interactive effect of freezing with other environmental drivers on plant functioning remains largely unexplored (but see Holdo, 2005, 2006, 2007). Our results show that four common savanna species from South African savannas are reasonably tolerant of freeze events, even at the seedling stage. Not only did most stems of all species survive three consecutive freeze events, but many leaves regained physiological functioning. Therefore, it seems unlikely that mild frosts, as would occur on the edge and just beyond the edge of the ranges of these species, would restrict the distribution of these species by preventing seedling establishment and growth.

### Do Dominant Savanna Tree Species Vary in their Tolerance to Water Limitation?

We assessed physiological tolerance to water limitation by measuring gas exchange and growth responses of plants grown under water-saturated and water-limited conditions. Considerable variation was found between the four species in terms of their responses to water limitation, which provides insights into how water availability may control their distributions, regardless of the influence of frost. The water availability treatments imposed were not meant to represent actual field conditions, but rather provided a continuum of water availability to assess differences in growth and physiology. The water-limited treatment significantly reduced RWC in the soil of all species by varying amounts (30–79%), which was likely attributed to differences in leaf area and amount of water used among species. We found that *C. mopane* was more tolerant to water limitation at both the leaf- and the whole-plant level than the other species in this experiment, which is consistent with its greater abundance in drier savannas, relative to the other three species. Neither leaf gas exchange rates nor growth parameters differed between water-limited and water-saturated *C. mopane*, while *A. nigrescens* and *C. abbreviata* each had reduced height, smaller stem diameters, and lower gas exchange rates when grown in the water-limited treatment.

We found that the ability of *C. mopane* to tolerate water limitation is likely related to its cellular and hydraulic properties. We assessed physiological drought-tolerance at the leaf level using leaf water potential at turgor loss (π_tlp_), which represents the water potential at which plant cells lose turgor and stomata close (Brodribb and Holbrook, 2003; Bartlett et al., 2012; Martorell et al., 2014). Leaves characterized by a low π_tlp_ have a large range over which they remain turgid and are typically able to maintain greater stomatal conductance and photosynthetic rates under drier conditions than leaves with a higher π_tlp_ (Sack et al., 2003; Bartlett et al., 2012). *Colophospermum mopane* had the lowest π_tlp_ in this experiment (**Table 1**), which suggests that this species can maintain physiological function under lower water potentials than the other species studied here and is consistent with our observations of gas exchange and growth responses to water limitation. Interestingly, *A. nigrescens* also had a low π_tlp_, despite showing large reductions in gas exchange rates with water stress (**Table 1**; **Figure 1**), suggesting that stomatal sensitivity in response to water limitation is decoupled from π_tlp_ in this species (Bartlett et al., 2012). Differences in stomatal sensitivity among *A. nigrescens* and *C. mopane* indicate that they have different water-use strategies (e.g., isohydric versus anisohydric) and suggest that these species may have differential responses to the future changes in precipitation predicted for this region (New et al., 2006). Understanding the physiological responses of *C. mopane* to climate change phenomena relative to other species will be critical, considering that this dominant species contributes greatly to ecosystem structure and function, (Timberlake et al., 2010), is an important browse species for elephants (Smallie and O’Connor, 2000), and is an economically important commodity for rural communities located throughout southern Africa (Makhado et al., 2014).

These results provide further support to explain the dominance of the Mopane savanna in arid and semi-arid areas relative to *Acacia–Combretum* savannas. *Colophospermum mopane* is thought to be more drought-tolerant than other woody species primarily because it can survive extreme drought stress (MacGregor and O’Connor, 2002) and has an extensive root system (Smit and Rethman, 1998), allowing it to grow in xeric locations (Henning and White, 1974) on a variety of soil types (Fraser et al., 1987). Water availability also regulates *C. mopane* and *A. nigrescens* distribution via impacts on seedling germination and establishment (Harrington, 1991; Stevens et al., 2014a), and is the primary factor initiating leaf drop during the dry season (Stevens et al., 2015). Despite these observations, no studies to our knowledge have explicitly tested the physiological drought tolerance of dominant savanna tree species in Mopane or *Acacia–Combretum* savannas.

### Do these Species Differ in their Sensitivity to Freezing Temperatures?

We hypothesized that *C. mopane* would show greater physiological susceptibility to freezing than the other three species which occur in areas that experience colder winters. Responses to nighttime freezing were similar for all four species, indicating that the distribution of *C. mopane* is unlikely to be limited by any increases in frequency of frost in areas of higher latitude or altitude. Furthermore, frost may not be a primary factor that excludes these four species from nearby grassland ecosystems that are only marginally colder during winter.

In our experiment we reduced nighttime temperatures to -5°C, a temperature that is rarely experienced within the range of the study species (Gertenbach, 1983; Kruger et al., 2002), and may not even occur regularly in colder areas just beyond their range. As the -5°C freeze events did not kill any of the seedlings, even under conditions of significant water limitation, our results suggest that frost is not important in restricting these species to their contemporary ranges. Not only did most stems survive across all species, many leaves survived the freeze events and continued to photosynthesize thereafter. Gas exchange rates decreased similarly for all species, but recovered fairly rapidly, particularly for *A. nigrescens* and *C. abbreviata.* Likewise, F_v_/F_m_ declined the most in *C. apiculatum* and the least in *A. nigrescens* and *C. abbreviata* (**Figure 3**), suggesting that *C. mopane* can tolerate freeze events at least as well as other species that occur farther south into colder regions. We also found that *C. mopane* leaf mortality was well within the range of leaf mortality experienced by all other species (Supplementary Table S6). Therefore, freezing temperatures alone do not likely explain the restricted distribution of *C. mopane* or the Mopane savanna across southern Africa. These findings support the work of Stevens et al. (2014b), who demonstrated that non-climatic factors, such as day length, are more important determinants of the regional *C. mopane* distribution than minimum temperatures, and that a southward *C. mopane* range expansion may not occur with future climate warming as previously predicted (Rutherford et al., 1999).

Furthermore, the observed recovery among most physiological parameters shows that successive nighttime freezing did not cause lasting responses and suggests that discrete frost events may not have long-term impacts on the function or survival of common tree seedlings from either the Mopane or the *Acacia–Combretum* savanna. Recovery among gas exchange parameters likely occurred because freezing did not reduce F_v_/F_m_ by more than 20% (**Figure 3**), and thus, did not impact the long-term photosynthetic potential of any species. The ability to regain photosynthetic function shortly after freezing may facilitate resprouting following partial stem mortality, which may effectively allow species to survive sporadically occurring frost disturbances (Ewers et al., 2003; Medeiros and Pockman, 2011). Although we did not measure resprouting in this experiment, our observation of physiological recovery despite partial leaf mortality suggests that these seedlings have the ability to continue carbon uptake and eventually resprout, which is consistent with previous reports of only partial mortality following freeze events in the field (Whitecross et al., 2012). This result is significant, considering the importance of tree recruitment (Holdo, 2005, 2007) and resprouting following disturbances in savannas (Higgins et al., 2000), as well as the presumed vulnerability of this demographic stage to freezing stress (Whitecross et al., 2012). However, we should note that nutrient limitations or more water-limited conditions might have produced different results. Also, it is currently unknown if freezing has an effect on the competitive ability of these species as they recover, or on other sensitive tissues such as buds, as has been observed in other species (Inouye, 2000; Charrier et al., 2013; Mayoral et al., 2015). Finally, the physiological responses to cold temperatures here reflect short-term exposure to absolute minimum temperature in the region (Gertenbach, 1983; Kruger et al., 2002), but it is still unclear how long-term exposure to ‘average’ minimum temperatures influences seedling physiology.

### Does Water Availability Modify Physiological Responses to Freezing?

Finally, we hypothesized that water limitation would exacerbate physiological responses to freezing because both stressors induce similar responses in plant hydraulics and because physiological responses to water availability have been shown to modify freezing sensitivity in a variety of other tree species (Ewers et al., 2003; Medeiros and Pockman, 2011; Charrier et al., 2015). The greater tolerance to freezing under limited water conditions was a unique response for *C. mopane* relative to the other three species. While this result is contrary to our prediction that *C. mopane* would be more sensitive to freezing when water-limited, it nevertheless suggests that a physiological link exists between plant responses to freezing and drought (Pratt et al., 2005). We found that leaf mortality was greater in water-saturated *C. mopane* compared to water-limited *C. mopane* (57 and 20% mortality, respectively; Supplementary Table S6). Of the remaining leaves, we observed leaf hydraulic conductivity loss in well-watered *C. mopane*, but this was not likely associated with the sensitivity to cavitation of xylem conduits experiencing low water potentials (Sperry, 1995; Davis et al., 1999) because hydraulic conductivity did not also decline in water-limited plants (**Figure 5**) despite both treatments having similar pre-freezing water potentials (**Figure 4**). We also did not observe greater hydraulic conductivity loss in species with water potentials lower than those observed in *C. mopane* (e.g., water-limited *A. nigrescens*; **Figures 4** and **5**), suggesting that hydraulic failure did not result from low water xylem potentials in those species either. We did observe corresponding declines in leaf water potential with freezing (**Figure 2**) but these values did not drop until after the third freeze event, indicating that water potential decreased in response to the decline in hydraulic conductivity.

Freezing-induced membrane damage, as indicated by electrolyte leakage, was also greater in water-saturated *C. mopane* than water-limited individuals (**Figure 6**) and was accompanied by damage to the photosynthetic apparatus (**Figure 3**) and a drop in photosynthesis (**Figure 2**). Water limitation can induce freezing tolerance in plants because osmotic adjustment, the accumulation of cellular solutes to maintain turgor pressure when water-limited (Morgan, 1984), may also reduce cellular dehydration as extracellular ice forms, thus reducing cellular membrane damage associated with freezing (Xin and Browse, 2000). Osmotic adjustment has been previously reported in osmotically stressed *C. mopane* (Johnson et al., 1996) and would explain the lower electrolyte leakage found among water-limited *C. mopane* in our study (**Figure 6**). Osmotic adjustment may also explain our hydraulic responses to freezing if membrane damage occurred in parenchyma cells that are involved with xylem refilling (Salleo et al., 2006; Brodersen et al., 2010; Nardini et al., 2011; Brodersen and McElrone, 2013). Damage to these cells would negatively affect the ability of water-saturated *C. mopane* to refill xylem conduits following freezing-induced cavitation (Charrier et al., 2013) and would consequently result in the greater decline of leaf hydraulic conductivity we observed among water-saturated plants compared to water-limited plants (**Figure 5**). Therefore, cellular responses to drought (e.g., osmotic adjustment) may reduce both leaf and xylem responses to freezing in *C. mopane*. These results are similar to previous observations that water stress reduced conductivity loss in *Larrea tridentata* (Medeiros and Pockman, 2011) and leaf damage in *Ceanothus* species (Ewers et al., 2003), although no studies to date have explicitly tested the relationship between drought, xylem refilling, and freezing. A more detailed investigation on the relationship between drought and freezing responses is warranted.

Water availability impacted physiological freezing responses to a lesser extent in other species. Electrolyte leakage did not manifest in *A. nigrescens* during freezing, and the leakage observed in *C. abbreviate* and *C. apiculatum* was greater in water-limited individuals (**Figure 6**), suggesting that osmotic adjustment during drought did not alleviate freezing damage in these species. Likewise, hydraulic responses to freezing did not differ among water treatments. Although midday leaf water potential was lower in water-limited *A. nigrescens* and *C. abbreviata*, these differences did not vary with freezing (**Figure 4**). Leaf hydraulic conductivity also did not vary with freezing for these species, although recovery values were greater in water-saturated plants (**Figure 5**). These results suggest that the link between drought and freezing responses is not consistent among species, which is not surprising considering the variability in both drought and freezing sensitivity observed among tree species and ecosystems globally (Pearce, 2001; Granda et al., 2014; Mayoral et al., 2015).

## Conclusion

Despite occurring in contrasting climates, tree species common to the Mopane and *Acacia–Combretum* savannas did not exhibit the distinct physiological responses to interactions of freezing and water availability initially expected. *Colophospermum mopane* was more tolerant to water limitation than the other species in this study, providing further support for the dominance of this species in drier climates compared to dominant species in the *Acacia–Combretum* savanna. However, all species were similarly sensitive to nighttime freezing, suggesting that freezing temperatures alone do not impact the distribution of these species. We also found that water availability modified select physiological responses to nighttime freezing events, and importantly, that water limitation actually improved freezing tolerance in one of the species (*C. mopane*). Ultimately, we show that unique physiologies can exist among dominant species within communities, these differences translate to novel responses to freezing and water availability, and that these combined stresses may play a currently unidentified role in driving the function and survival of these species across southern Africa.

## Author Contributions

JN and KO conceived and designed the experiment. KO performed the experiment and analyzed the data. KO, JN, and AS wrote the manuscript.

## Conflict of Interest Statement

The authors declare that the research was conducted in the absence of any commercial or financial relationships that could be construed as a potential conflict of interest.
