# Supplementary material for: Savanna Tree Seedlings are Physiologically Tolerant to Nighttime Freeze Events
Source: Front Plant Sci. 2016 Feb 2;7:46. doi: 10.3389/fpls.2016.00046 (PMC4735699; doi:10.3389/fpls.2016.00046)
Supplement: Supplementary file 1 [file Data_Sheet_1.PDF]

Supplementary Material

Savanna Tree Seedlings are Physiologically Tolerant to Nighttime Freeze Events

Kimberly O'Keefe*, Jesse B. Nippert, Anthony M. Swemmer

*Correspondence: Kimberly O'Keefe: kokeefe@ksu.edu

Supplementary Tables

Supplementary Table 1. The relative water content (RWC) of soil measured in each pot. Shown are mean ± 1 SEM measured for all species and water treatments, on each sampling date (days since germination). RWC was measured in units of water fraction by volume (wfv).

Date	*C. mopane*		*A. nigrescens*		*C. abbreviata*		*C. apiculatum*	
	Water-saturated	Water-limited	Water-saturated	Water-limited	Water-saturated	Water-limited	Water-saturated	Water-limited
148	0.22 ± 0.01	0.04 ± 0.01	0.11 ± 0.03	0.002 ± 0.002	0.11 ± 0.02	0.00 ± 0.00	0.19 ± 0.01	0.10 ± 0.02
163	0.30 ± 0.01	0.14 ± 0.02	0.24 ± 0.01	0.03 ± 0.01	0.24 ± 0.02	0.04 ± 0.01	0.28 ± 0.01	0.17 ± 0.02
184	0.29 ± 0.01	0.19 ± 0.01	0.24 ± 0.01	0.09 ± 0.01	0.19 ± 0.03	0.09 ± 0.01	0.28 ± 0.02	0.20 ± 0.01
198	0.24 ± 0.01	0.10 ± 0.01	0.17 ± 0.01	0.04 ± 0.01	0.16 ± 0.13	0.01 ± 0.01	0.26 ± 0.02	0.14 ± 0.01
219	0.31 ± 0.01	0.07 ± 0.01	0.20 ± 0.01	0.01 ± 0.003	0.22 ± 0.02	0.01 ± 0.01	0.30 ± 0.01	0.23 ± 0.02
233	0.29 ± 0.01	0.16 ± 0.01	0.20 ± 0.01	0.04 ± 0.01	0.18 ± 0.01	0.08 ± 0.01	0.27 ± 0.01	0.20 ± 0.01
247	0.30 ± 0.01	0.12 ± 0.01	0.12 ± 0.01	0.02 ± 0.01	0.07 ± 0.03	0.04 ± 0.01	0.26 ± 0.01	0.19 ± 0.01
260	0.30 ± 0.01	0.09 ± 0.01	0.11 ± 0.01	0.01 ± 0.004	0.11 ± 0.02	0.003 ± 0.002	0.26 ± 0.02	0.19 ± 0.02
275	0.31 ± 0.01	0.17 ± 0.01	0.17 ± 0.01	0.06 ± 0.01	0.19 ± 0.02	0.09 ± 0.03	0.29 ± 0.01	0.25 ± 0.02

Supplementary Table 2. Linear mixed-effects model of plant growth and soil moisture responses to water treatments. Species, water treatment, and sample day as fixed effects are fixed effects and plant was included as a random effect. Shown are *F*- and *P*-values for stem height, stem diameter, and relative water content (RWC). Significance is indicated at the α=0.05 level with an asterisk (*).

		**Height**	**Diameter**	**RWC**
**Species**	*F*	33.07	384.47	139.91
	P	< 0.0001*	< 0.0001*	< 0.0001*
**Water**	*F*	14.27	39.80	427.56
	P	0.0002*	< 0.0001*	< 0.0001*
**Day**	*F*	54.00	106.67	34.74
	P	< 0.0001*	< 0.0001*	< 0.0001*
**Species*Water**	*F*	0.74	9.91	9.93
	P	0.5292	< 0.0001*	< 0.0001*
**Species*Day**	*F*	6.41	19.83	5.71
	P	< 0.0001*	< 0.0001*	< 0.0001*
**Water*Day**	*F*	24.85	29.23	9.91
	P	< 0.0001*	< 0.0001*	< 0.0001*
**Species*Water*Day**	*F*	4.71	8.32	4.24
	P	< 0.0001*	< 0.0001*	< 0.0001*

Supplementary Table 3. Three-way ANOVA of plant physiological responses to water treatments. Species, water treatment, and sampling date are fixed effects. Shown are *F*- and *P*-values for CO_2 assimilation (A_{max}), stomatal conductance (g_s), and transpiration rate (*E*). Significance is indicated at the α=0.05 level with an asterisk (*).

		A_{max}	g_s	*E*
**Species**	*F*	15.67	12.58	10.96
	P	< 0.0001*	< 0.0001*	< 0.0001*
**Water**	*F*	103.99	107.33	104.55
	P	< 0.0001*	< 0.0001*	< 0.0001*
**Day**	*F*	2.31	5.73	23.93
	P	0.1295	0.0174*	< 0.0001*
**Species*Water**	*F*	11.40	20.34	17.53
	P	< 0.0001*	< 0.0001*	< 0.0001*
**Species*Day**	*F*	1.37	0.73	0.38
	P	0.2514	0.5362	0.7710
**Water*Day**	*F*	2.70	0.93	3.64
	P	0.1016	0.3355	0.0575
**Species*Water*Day**	*F*	2.38	2.01	1.60
	P	0.0704	0.1127	0.1899

Supplementary Table 4. Species physiological responses to water treatments prior to freezing. Mean ± 1 SEM stomatal conductance of water vapor, g_s (mol $m^{-2}s^{-1}$) and transpiration rate, *E* (mmol $m^{-2}s^{-1}$) are shown for all species and water treatments, on each sampling date (days since germination).

Date	*C. mopane*		*A. nigrescens*		*C. abbreviata*		*C. apiculatum*	
	Water-saturated	Water-limited	Water-saturated	Water-limited	Water-saturated	Water-limited	Water-saturated	Water-limited
g_s								
155	0.07 ± 0.01	0.05 ± 0.01	0.11 ± 0.02	0.05 ± 0.01	0.12 ± 0.02	0.04 ± 0.01	0.05 ± 0.01	0.03 ± 0.01
190	0.02 ± 0.01	0.02 ± 0.01	0.09 ± 0.02	0.02 ± 0.01	0.02 ± 0.01	0.01 ± 0.001	0.03 ± 0.004	0.02 ± 0.003
216	0.09 ± 0.01	0.07 ± 0.01	0.14 ± 0.02	0.02 ± 0.003	0.08 ± 0.004	0.02 ± 0.004	0.02 ± 0.003	0.02 ± 0.003
274	0.07 ± 0.01	0.07 ± 0.01	0.14 ± 0.02	0.04 ± 0.01	0.08 ± 0.01	0.05 ± 0.004	0.05 ± 0.01	0.05 ± 0.01
E								
155	1.71 ± 0.21	1.35 ± 0.13	2.95 ± 0.50	1.24 ± 0.35	2.60 ± 0.33	1.06 ± 0.22	1.46 ± 0.26	0.86 ± 0.21
190	0.49 ± 0.12	0.51 ± 0.13	2.07 ± 0.50	0.38 ± 0.20	0.48 ± 0.11	0.16 ± 0.03	0.56 ± 0.01	0.42 ± 0.06
216	3.11 ± 0.29	2.69 ± 0.47	4.76 ± 0.49	0.86 ± 0.14	3.28 ± 0.17	0.85 ± 0.20	2.27 ± 0.25	0.61 ± 0.10
274	1.86 ± 0.19	1.71 ± 0.19	4.07 ± 0.38	1.29 ± 0.18	2.06 ± 0.29	1.46 ± 0.11	2.30 ± 0.32	1.45 ± 0.17

Supplementary Table 5. Stem height (cm) and stem diameter (mm) responses to water treatments, prior to freezing. Shown are mean ± 1 SEM measured for all species and water treatments, on each sampling date (days since germination).

Date	*C. mopane*		*A. nigrescens*		*C. abbreviata*		*C. apiculatum*	
	**Water-saturated**	**Water-limited**	**Water-saturated**	**Water-limited**	**Water-saturated**	**Water-limited**	**Water-saturated**	**Water-limited**
**Height**								
148	47.52 ± 2.58	46.35 ± 2.19	58.46 ± 1.89	59.09 ± 1.44	35.43 ± 2.54	35.19 ± 1.69	50.25 ± 4.08	42.92 ± 4.00
163	49.90 ± 2.65	46.87 ± 2.48	61.93 ± 1.89	61.47 ± 1.43	37.43 ± 2.19	34.75 ± 1.32	54.27 ± 4.13	44.21 ± 3.88
184	51.49 ± 2.76	48.50 ± 2.30	66.28 ± 1.86	61.82 ± 1.49	39.86 ± 1.82	35.40 ± 1.38	53.42 ± 4.55	44.71 ± 4.03
198	52.97 ± 2.93	49.56 ± 2.33	71.03 ± 1.71	62.65 ± 1.42	46.77 ± 2.31	35.20 ± 1.35	53.45 ± 4.43	44.48 ± 3.96
219	51.87 ± 2.83	49.28 ± 2.25	68.32 ± 1.74	61.86 ± 1.48	45.04 ± 2.90	35.30 ± 1.35	53.23 ± 4.39	42.97 ± 3.68
233	53.62 ± 2.89	50.14 ± 2.26	69.79 ± 1.82	62.14 ± 1.53	49.91 ± 3.28	36.23 ± 0.96	54.48 ± 4.44	43.78 ± 4.13
247	54.07 ± 3.04	49.44 ± 2.27	76.09 ± 1.69	62.93 ± 1.56	52.17 ± 4.84	35.49 ± 1.29	55.57 ± 4.09	44.87 ± 4.13
260	55.81 ± 2.97	50.99 ± 2.25	75.77 ± 1.65	63.54 ± 1.56	57.20 ± 6.18	36.79 ± 1.59	53.37 ± 3.90	45.77 ± 4.17
275	55.42 ± 2.92	50.03 ± 2.29	75.60 ± 1.83	63.38 ± 1.50	58.07 ± 6.14	36.81 ± 1.65	52.83 ± 3.78	41.70 ± 4.34
**Diameter**								
148	5.59 ± 0.21	5.81 ± 0.19	10.10 ± 0.17	10.07 ± 0.21	5.39 ± 0.43	4.97 ± 0.31	4.29 ± 0.32	3.12 ± 0.33
163	6.34 ± 0.26	6.19 ± 0.21	11.46 ± 0.26	11.01 ± 0.22	5.78 ± 0.30	5.34 ± 0.36	4.71 ± 0.30	3.57 ± 0.31
184	6.01 ± 0.25	6.37 ± 0.24	12.07 ± 0.33	10.72 ± 0.22	6.22 ± 0.27	5.27 ± 0.35	4.63 ± 0.27	3.36 ± 0.33
198	6.38 ± 0.26	6.74 ± 0.25	12.47 ± 0.33	10.82 ± 0.25	6.96 ± 0.45	5.21 ± 0.32	4.78 ± 0.33	3.67 ± 0.38
219	6.30 ± 0.26	6.63 ± 0.26	13.29 ± 0.31	11.45 ± 0.24	7.94 ± 0.28	5.49 ± 0.29	5.01 ± 0.41	3.90 ± 0.52
233	6.44 ± 0.25	6.55 ± 0.21	14.05± 0.35	11.61 ± 0.19	8.22 ± 0.32	5.47 ± 0.37	5.31 ± 0.43	3.69 ± 0.44
247	6.75 ± 0.28	6.77 ± 0.24	14.94 ± 0.34	11.72 ± 0.21	9.25 ± 0.49	5.53 ± 0.29	5.53 ± 0.47	3.41 ± 0.43
260	7.27 ± 0.32	7.57 ± 0.27	15.68 ± 0.33	12.16 ± 0.26	9.44 ± 0.57	5.58 ± 0.29	5.46 ± 0.44	3.26 ± 0.46
275	7.29 ± 0.35	7.57 ± 0.25	16.43 ± 0.48	12.65 ± 0.19	9.32 ± 0.39	5.46 ± 0.24	5.71 ± 0.39	3.27 ± 0.38

Supplementary Table 6. Mean ± 1 SEM percent leaf death recorded for each species one week following the last freeze event and statistics assessing differences in leaf death among species. *F* and *P* values are calculated from a linear mixed effects model where species and water treatment are fixed effects and freezing sequence replicate is a random effect. Significance is indicated at the α=0.05 level with an asterisk (*).

**Species**	**Water-saturated**	**Water-limited**
C. mopane	57.14 ± 11.07	20.00 ± 10.24
A. nigrescens	10.00 ± 2.18	15.00 ± 3.07
C. abbreviata	65.71 ± 13.60	52.86 ± 13.40
C. apiculatum	36.43 ± 14.09	61.25 ± 12.31
**Statistics**	***F***	***P***
Species	8.71	0.0001*
Water	0.54	0.4632
Species x Water	3.28	0.0308*

Supplementary Table 7. Linear mixed-effects model of physiological responses to freezing. Species, water treatment, and freezing day are fixed effects, and plant and freezing sequence replicate are included as random effects. Shown are *F*- and *P*-values for CO_2 assimilation (A_{max}), stomatal conductance (g_s), transpiration rate (*E*), photosynthetic efficiency (F_v/F_m), midday leaf water potential (Ψ_{leaf}), leaf hydraulic conductivity (K_{leaf}), and electrolyte leakage (EL). Significance is indicated at the α=0.05 level with an asterisk (*).

		A_{max}	g_s	E	F_v/F_m	Ψ_{leaf}	K_{leaf}	EL
**Species**	*F*	14.55	10.61	6.05	8.57	3.68	11.89	12.45
	P	< 0.0001*	< 0.0001*	0.0006*	0.0001*	0.0198*	< 0.0001*	< 0.0001*
**Water**	*F*	25.47	22.27	19.91	0.001	10.36	0.01	5.02
	P	< 0.0001*	< 0.0001*	< 0.0001*	0.9791	0.0025*	0.9226	0.0306*
**Day**	*F*	182.03	120.62	88.57	11.03	4.91	9.14	2.96
	P	< 0.0001*	< 0.0001*	< 0.0001*	< 0.0001*	0.0029*	< 0.0001*	0.0347*
**Species*Water**	*F*	9.86	12.06	9.40	0.78	7.30	4.64	4.16
	P	< 0.0001*	< 0.0001*	< 0.0001*	0.5123	0.0005*	0.0038*	0.0118*
**Species*Day**	*F*	6.30	4.57	4.30	2.25	2.60	1.32	1.02
	P	< 0.0001*	< 0.0001*	< 0.0001*	0.0229*	0.0085*	0.2325	0.4230
**Water*Day**	*F*	13.82	7.31	5.53	1.14	2.20	1.56	1.66
	P	< 0.0001*	0.0012*	0.0012*	0.3345	0.0912	0.2001	0.1783
**Species*Water*Day**	*F*	3.53	3.63	2.67	0.29	1.77	0.55	1.03
	P	0.0006*	0.0035*	0.0063*	0.9762	0.0798	0.8377	0.4219
